# A Miniaturized and Highly Sensitive Microwave Sensor Based on CSRR for Characterization of Liquid Materials

**DOI:** 10.3390/ma16093416

**Published:** 2023-04-27

**Authors:** Ahmed Jamal Abdullah Al-Gburi, Zahriladha Zakaria, Norhanani Abd Rahman, Ayman A. Althuwayb, Imran Mohd Ibrahim, Tale Saeidi, Zaheer Ahmed Dayo, Sarosh Ahmad

**Affiliations:** 1 Centre for Telecommunication Research & Innovation (CeTRI), Faculty of Electrical and Electronic Engineering Technology (FTKEE), Malacca 76100, Malaysia; ahmedjamal@utem.edu.my; 2 Centre for Telecommunication Research & Innovation (CeTRI), Fakulti Kejuruteraan Elektronik dan Kejuruteraan Komputer (FKEKK), Universiti Teknikal Malaysia Melaka, Durian Tungal 76100, Malaysia; 3 Department of Electrical Engineering, Politeknik Port Dickson (PPD), Port Dickson 71250, Malaysia; 4 Electrical Engineering Department, Engineering College, Jouf University, Sakaka 72388, Saudi Arabia; aaalthuwayb@ju.edu.sa; 5 Electrical and Electronics Engineering Department, Faculty of Engineering and Natural Sciences, İstinye University, Istanbul 34396, Turkey; 6 College of Computer Science, Huanggang Normal University, Huangzhou 438000, China; 7 Department of Signal Theory and Communications, Universidad Carlos III de Madrid (UC3M), 28911 Madrid, Spain

**Keywords:** complementary split-ring resonator (CSRR), liquid media under tests (MUTs), triple rings (TRs), sensitivity, Q-factor, polypropylene (PP) tube, ethanol, methanol, distilled water (DI)

## Abstract

In this work, a miniaturized and highly sensitive microwave sensor based on a complementary split-ring resonator (CSRR) is proposed for the detection of liquid materials. The modeled sensor was designed based on the CSRR structure with triple rings (TRs) and a curve feed for improved measurement sensitivity. The designed sensor oscillates at a single frequency of 2.5 GHz, which is simulated using an Ansys HFSS simulator. The electromagnetic simulation explains the basis of the mode resonance of all two-port resonators. Five variations of the liquid media under tests (MUTs) are simulated and measured. These liquid MUTs are as follows: without a sample (without a tube), air (empty tube), ethanol, methanol, and distilled water (DI). A detailed sensitivity calculation is performed for the resonance band at 2.5 GHz. The MUTs mechanism is performed with a polypropylene tube (PP). The samples of dielectric material are filled into PP tube channels and loaded into the CSRR center hole; the E-fields around the sensor affect the relationship with the liquid MUTs, resulting in a high Q-factor value. The final sensor has a Q-factor value and sensitivity of 520 and 7.032 (MHz)/*ε_r_*) at 2.5 GHz, respectively. Due to the high sensitivity of the presented sensor for characterizing various liquid penetrations, the sensor is also of interest for accurate estimations of solute concentrations in liquid media. Finally, the relationship between the permittivity and Q-factor value at the resonant frequency is derived and investigated. These given results make the presented resonator ideal for the characterization of liquid materials.

## 1. Introduction

In recent years, there has been a rapid development of interest in microwave resonator sensors for various technological challenges, such as the detection of the properties of media under tests (MUT) via structural analyses. Microwave sensors are among the most widely used sensors for material characterization in agriculture, pharmaceuticals, and industry [1,2,3]. Material characterization is necessary when one wants to distinguish the type of material for a particular application. It may be a solid, semi-solid, or powder sample [4,5,6]. Sensor sensitivity is also crucial for microwave engineering when it comes to the properties of the characterized materials. Compared to solid materials, the dielectric constant of liquid materials is more likely to be affected by aspects such as temperature, humidity, impurities in the sample holder, atmospheric pressure, and others [7,8,9]. In addition, experiments with a liquid sample are less suitable due to its fluid behavior. Chemical liquid samples consist of polar particles that have a high dielectric constant and dielectric loss. Therefore, the development of in situ dielectric constant and loss tangent experiments for liquid materials represents a dynamic market [10].

Microwave resonator sensors can be either passive or active devices with a single- or double-port network transmission line that relays the desired electromagnetic wave signal. It was found that planar microwave sensors with a microstrip split-ring resonator (SRR) and circular substrate-integrated waveguide topologies were capable of producing high Q-factors and sensitivities. The dielectric properties of the materials and the sensitivity of the resonator based on perturbation theory were measured accordingly. In addition, a wide range of resonators with different specifications were tested, including the resonant frequency, Q-factor, and bandwidth, and researchers were able to determine the material quantities and dielectric characterization of solid, semisolid, and liquid materials in real time. This provided a new, highly suitable method for determining dielectric properties in pharmaceutical, subsurface, chemical, biological, and biomedical applications [11,12,13,14]. Material characterization measurements are typically performed by analyzing the resonant frequencies of the sensors used to detect material properties. These resonant frequencies can be classified into two types: resonators, which are designed to oscillate at specific frequencies and are highly sensitive to changes in a material’s physical properties; and disturbances, which occur when a material exhibits significant changes in its mechanical behavior. According to previous studies [15,16], these two types of resonant frequencies can provide complementary information about a material’s microstructure and macroscopic properties, making them useful for a wide range of applications. Resonance methods can accurately describe material properties with a high sensitivity at specific resonant frequencies, in contrast to broadband techniques that provide information across a range of frequencies. Microwaves, insulating materials, and coaxial resonators are versatile tools that are widely used in materials science and engineering. They can be used to analyze the physical and chemical properties of materials in various settings, such as high temperatures, high pressures, or corrosive environments [17,18,19]. These resonators have been developed to meet the needs of industry as they are suitable for measuring materials and have a high accuracy. The various dielectric properties of substrates can be detected by a resonator in terms of its reflection and transmission coefficient characteristics [20,21,22]. However, attaining a good performance for microwave sensors in dielectric and fluid measurements for material detection is still a challenge. Conversely, these resonators could be more complicated in fabrication and have complex relative permittivity and permeability. Planar microwave sensors are used in this case due to their numerous features, such as having low costs, simple structures, and real-time characterization capabilities, as well as the ease of their fabrication [23,24,25,26].

In materials characterization, microwave planar sensors offer the advantages of measurements in a wide range of samples. They have a minimal error tolerance for material characterization due to their ability to characterize materials over a high Q-factor, as described in [27,28]. Non-planar resonator sensors offer unique advantages for sensing applications, but working with these sensors presents some challenges. Specifically, their complex geometries make it difficult to use conventional characterization techniques, leading to high fabrication costs and a large number of required measurements and experimental procedures. Additionally, the size of these devices can limit their use in critical applications [29,30,31,32].

Numerous experimental setups and measurement techniques have been proposed in the literature to characterize liquid samples for different concentrations, such as measuring the dielectric constant [33]. One of the most common sensors used for liquid detection is the coaxial probe, which requires immersion in the measured samples and may not be suitable for all applications [34,35,36,37]. However, noninvasive sensing methods have been developed for human health monitoring, including a dual-band sensor that can detect glucose levels [38] and human milk samples [39]. Two different methods have been presented to characterize the permittivity of the sensor: dumbbell-shaped microwave sensors [40] and slow-wavelength microfluidic integrated waveguides [41]. In particular, numerous approaches have been used to determine the properties of the samples under study, such as complementary split-ring resonators (CSRRs), which are considered to be the most typical resonators in the structure of microwave sensors for fluids [42,43,44,45]. In [42], a sensor was designed to maintain the media under tests (MUTs) in a liquid state during experiments, which caused an RF loss in the liquid and affected the measurement accuracy. While the microwave sensors proposed by Kiani et al. [43] can accurately measure the dielectric constant of liquids, they are unable to measure the loss tangent of the liquid material. The sensor proposed by Su et al. [44] uses flexible fabrics but is limited in its use with low-loss materials due to its sensitivity. Meanwhile, the sensitivity of the sensor described in Ref [45] is too low at only 150 MHz/(mgmL), and a high level of noise was observed throughout the measurement process, limiting its utility for many applications.

In this work, the sensor was simulated, tested, and measured using a mixture of ethanol, methanol, and distilled water. The motivation to test ethanol stems from its high concentration and use in chemical environments. Under these circumstances, sensors must be highly sensitive to accurately monitor the ethanol concentration. The resonant frequency of ethanol at various concentrations from 10% to 100% has been clearly discussed. The proposed sensor is miniaturized with a size of about 25 mm × 20 mm, which makes it low profile compared to [46]. The modeled sensor has a high sensitivity of about 7.321 (MHz)/*ε_r_*) at 2.5 GHz. Through detailed analyses and experiments, the presented resonator can identify the topologies of liquid MUTs and detect their concentrations according to the proposed sensors [47,48]. The proposed sensor has proven that it can be operated with a high sensitivity and lower concentrations of ethanol and DI water; the proposed CSSR sensor has a lower concentration limit than 10% ethanol.

## 2. Sensor Configuration and Liquid MUTs Validation

### 2.1. Sensor Configuration

Initially, the modeled sensor configuration was developed based on the conventional geometry of the CSRR described in [49], and the idea of the implemented sensor was presented by [50,51]. It was found that the CSRR loop provides a more appropriate sensitivity in contrast to the elongated CSRR, which maintains the exact unit size. Similarly, the curve feed design configuration was carefully conducted by the authors step-by-step as suggested in [6]. A Rogers RT Duroid 6002 laminate with a dielectric loss of 0.0012 was selected for the design process.

**Figure 1:**
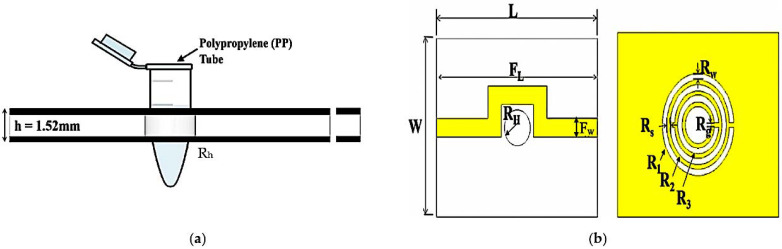
CSRR TRs sensor design structure; (**a**) Side view of TR structure with center hole to place PP tube; (**b**) Top view of the transmission line position, where: *L* = 25mm, *W* = 20 mm, *F_L_* = 28 mm, *R_H_* = 2 mm, and *F_W_* = 2.1 mm, *R_W_* = 0.68 mm, *R_S_* = 0.5 mm, *R_g_* = 0.5 mm, *R_1_* = 5.54 mm, *R_2_* = 3.18 mm, and *R_3_* = 3.18 mm.

A 0.5 mm coupling gap was chosen to support the circular CSRR design geometry, which is crucial to selecting the capacitance energy gap of the circular design. In contrast, the current distribution near the rings induced electric and magnetic fields due to the patch properties. The dimensions of the triple rings’ (TRs) sensor in Figure 1 are 25 mm × 20 mm × 1.52 mm (L × W × H). To optimize the performance, the TR sensor is embedded in a 2 mm diameter PP tube with a dielectric constant of 2.1 as the liquid MUTs channel.

**Figure 2:**
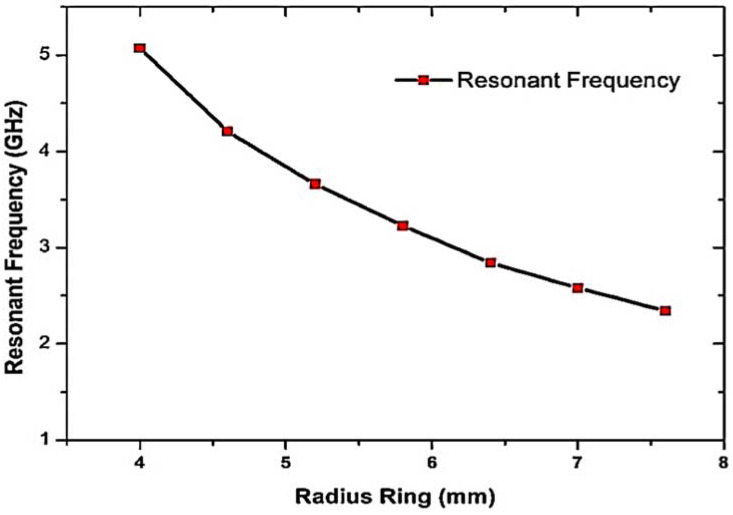
The effect of the resonant frequency of CSRR sensor with rings of various radii.

To investigate the effects of the SRRs’ size with respect to the total radius, ANSYS HFSS software was used to determine the S-parameters for an SRR unit cell. In this parametric analysis, the width of the copper trace and the dielectric height (h) were held constant. In addition, the dimensions of the cell’s confining unit were fixed (L and W). Only the gap size (R_g_) and radius were varied (R_1_, R_2_, and R_3_). This changes the overall diameter of the ring and thus its overall size. Starting from the standard resonant frequency, the resonant frequency decreases as both the capacitance and inductance are increased. The simulation outcomes are presented in Figure 2. 

**Figure 3:**
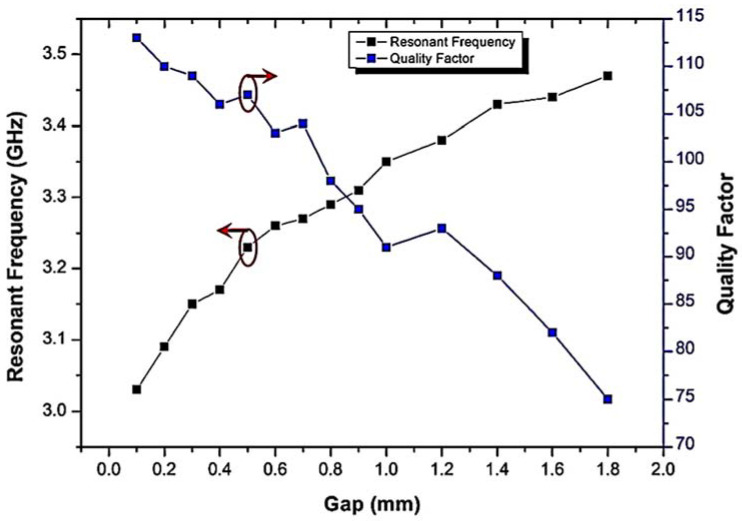
The resonant frequency and Q-factor of CSRR for varying the ring gap.

Another interesting parameter is the gap of the ring. The same analysis was applied to the ring gap by choosing an outer radius (R_1_) of 5.54 mm. It can be seen that an expansion in the ring gap leads to a high inductance value and causes a reduction in the Q-factor. As a result, the resonant frequency increases exponentially with the ring gap, as depicted in Figure 3. The Q-factor of the annular gap has a high value for the initial gap of 0.1 mm and then decreases. This shows that a small gap produces a high Q-factor. However, a high inductance must be created in the CSRR structure to generate strong electric fields between the gap and the slot of the ring. In addition, choosing a gap that is too narrow can lead to a manufacturing problem. Therefore, the dimension of the gap Rg = 0.5 mm with a Q-factor of 107 at 3.23 GHz is very suitable.

**Figure 4:**
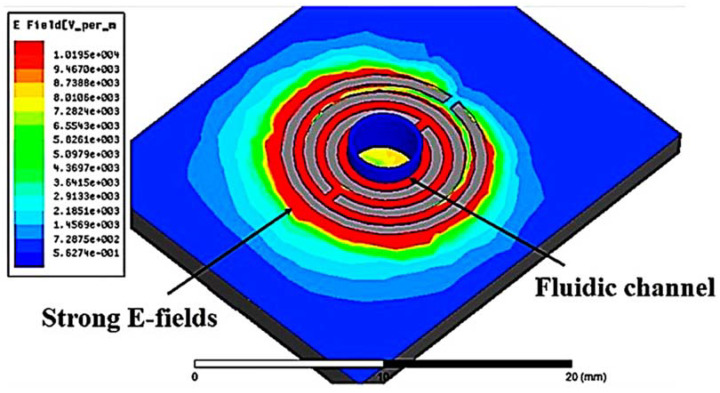
Location of a fluid channel and the magnitude of the electric field distribution around the channel.

The principle of detecting changes in liquid MUT properties works through tube loading at the center of the sensor. Figure 4 describes the maximum exposure of the electric fields around the tube. There are one-shape elements at the feed line around the hole for electromagnetic excitation when the microfluidic channel is loaded at the center. 

Several liquid MUTs were analyzed and experimented with the proposed TRs sensor. The chamber temperature was kept constant to avoid unwanted disturbances during the experiments. Due to the electromagnetic effects of the samples, their induced shift in the CSRR’s resonant frequency, insertion loss (S21), and Q-factor differ slightly.

### 2.2. Liquid Media under Tests (MUTs)

**Figure 5:**
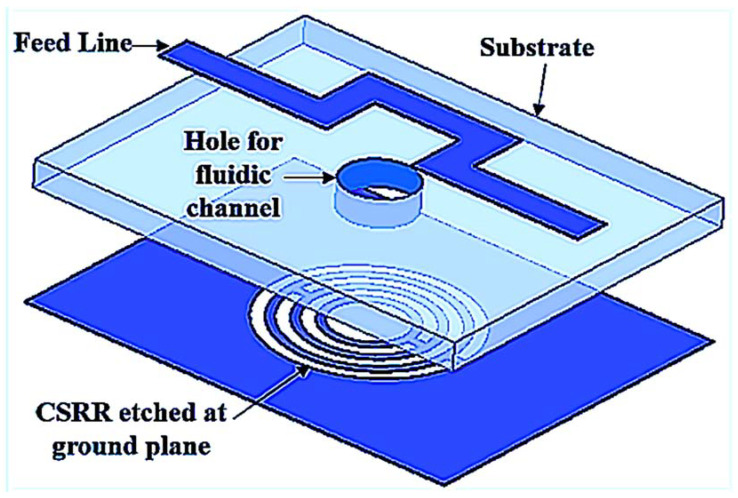
Perspective view of TR CSRR sensor.

The liquid MUTs are discussed and demonstrated in this subsection. The designed TR CSRR sensor is etched at the ground plane with a curve U-shaped feed line to drop strong electromagnetic excitation around the hole, as shown in Figure 5. In order to prove the concept of our design, numerous drop tests were conducted by testing the influence of the liquid MUT on the sensors’ transmission properties. 

**Figure 6:**
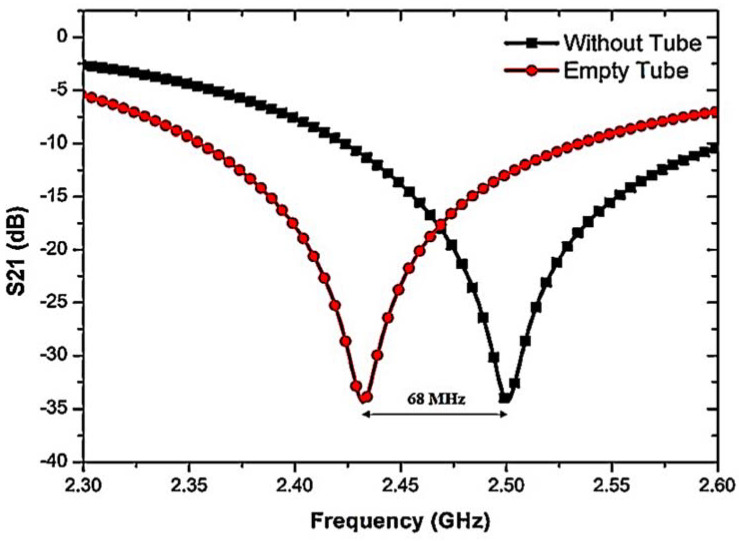
The effect of frequency shift when the PP tube is loaded into the sensor.

Another important analysis for characterizing liquid MUTs is the use of a polypropylene (PP) tube. Samples of dielectric material are filled into the channel of the PP tube and loaded into the CSRR center hole. The E-fields near the resonator affect the interaction with the liquid MUTs. Our observations were that the resonant frequency shifts slightly to a lower frequency of 2.432 GHz with a shift of 68 MHz when the empty tube is loaded (see Figure 6).

**Figure 7:**
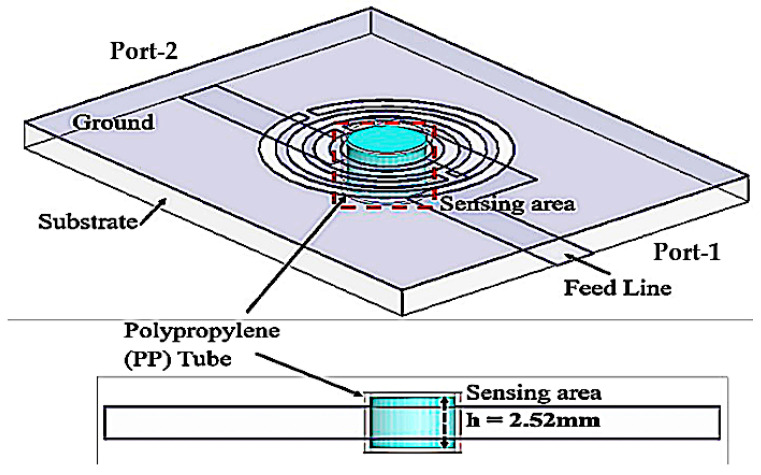
PP tube loaded into the substrate for 7.92 μL at a time.

To evaluate the sensing area of the tube, we characterize the sample volume based on the sensor thickness and the location of the maximum electric flux. The volume is calculated using Equation (1) and is illustrated in Figure 7, which shows a close-up image of the sensing region. The best performance is achieved by using tube volume lengths such that the average frequency change exceeds a single saturation level (h).

𝑉 = 𝜋𝑟^2^h (1)

where *r* is the radius of the fluidic channel and h is the height of the sensing area based on the saturation level of volume. 

**Figure 8:**
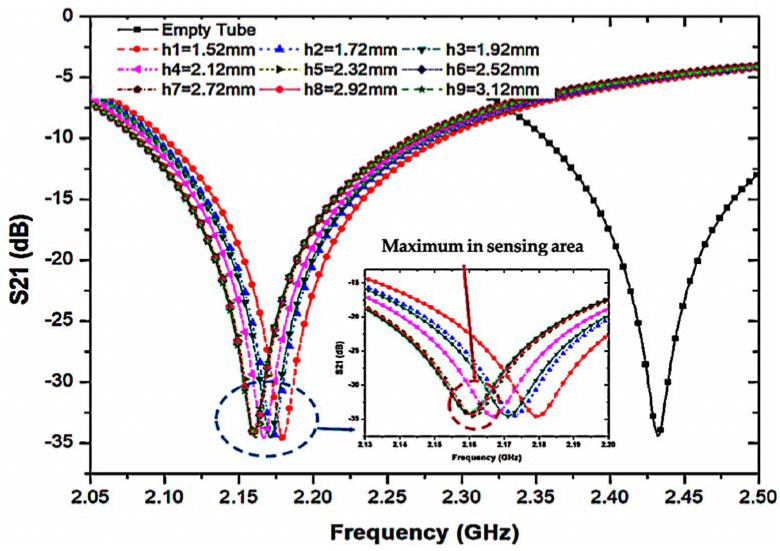
Simulation of the frequency change in different volume levels of the sensing region utilizing distilled water filled in the PP tube.

The simulated transmission coefficient (S21) of the suggested sensor with the empty and distilled water (DI water) loaded into a 6 mm tube as indicated in Figure 8, where the optimal volume length is 2.52 mm, equivalent to 7.92 μL of liquid. 

**Figure 9:**
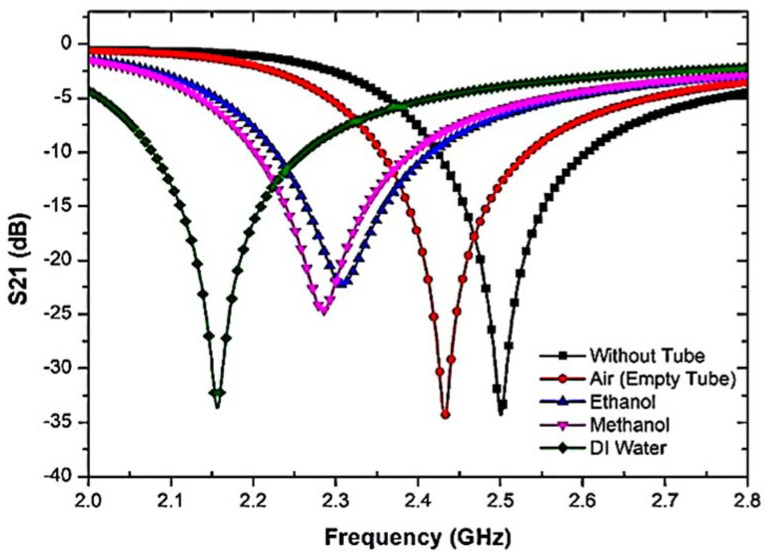
The frequency response of TR CSRR sensor with liquid MUTs samples.

The resonant frequency was also measured in the presence and absence of liquid MUTs. Each sample has dielectric effects that interact with the electric fields in the sensor region and are eventually clarified as a response to the characterization of the properties. Figure 9 shows that the resonant frequency and insertion loss were significantly changed due to the polar properties of the samples.

To further study the response of the sensor to the TR CSRR sensor, different fluid types with different dielectric characteristics and relaxation periods were used. Each sample has dielectric properties that perturb the electric fields in the sensor region and are ultimately described as a response to the characterization of the properties. Figure 9 shows that due to the polar properties of the samples, the resonant frequency and insertion loss were obviously changed.

The analysis of the two connected networks recognized the importance of the interference response and the transmitted information in determining the dielectric properties [52]. The typical dielectric constants for liquid specimens of water, methanol, and ethanol are 𝜀′ of 78.4, 32.7, and 24.5, respectively [53]. Furthermore, the permittivity value is used in [54]. Regarding the dielectric properties of the present samples, the quality factor of the compact resonator sensors was reduced. Table 1 illustrates the outcomes of the frequency response analysis when liquid MUTs are utilized.

The temperature is monitored consistently, and various sample tests are standardized to obtain accurate average test values. To ensure consistency with the theoretical principle, slight frequency deviations are detected and critically compared with the measured data at a temperature of 0.05 degrees.

### 2.3. Ethanol Concentration

**Figure 10:**
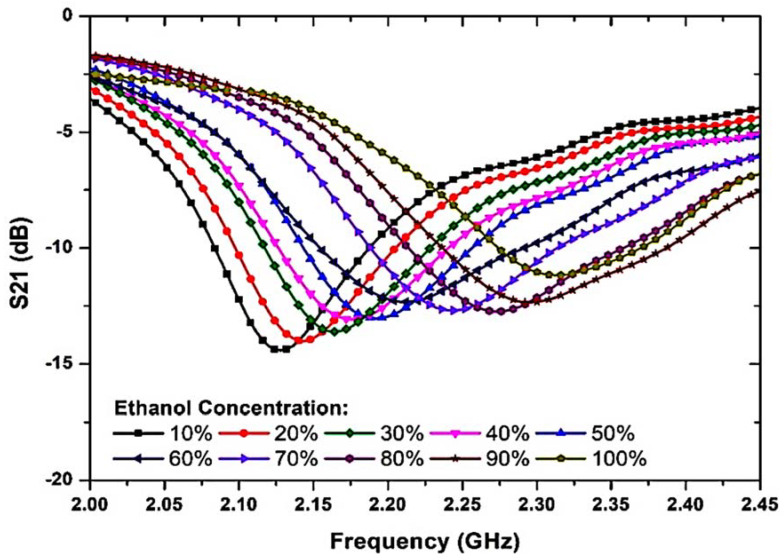
Measured S-parameters’ ethanol concentrations.

The response to variations in ethanol concentration is illustrated in Figure 10 and Table 2, showing that the ethanol concentration among the volume fraction of water varied from 10% to 100% with a step size of 10%. This characterization method describes the tendency within this specific mixture and can be used to evaluate the composition of any distilled water–ethanol (C_2_H_8_O_2_) mixture. The maximum shift spans a range of 200 MHz. The concentration level of the MUT mixture shows a non-overlapping resonant frequency that allows for a realistic variance in the concentration using the proposed sensor. The relationship between the resonant frequency and the ethanol concentration is close to y = 8× 10^−6^𝑓^2^ − 0.0012𝑓 + 2.1152 from 10% to 100% concentrations.

The suggested sensor’s lower detection limit is 10% ethanol. To develop 10% ethanol, 0.2 mL of DI water was combined with 1.8 mL of 100% ethanol. The absolute ethanol solvent (C_2_H_6_O, part number 19,535) was manufactured by Chemiz (M) Sdn. Bhd., Malaysia. The resonance frequency was reduced from 2.312 GHz to 2.112 GHz, as the ethanol concentration increased from 0% to 100%. It was observed that the proposed sensor showed a 200 MHz frequency shift for the ethanol concentration, which is better than that of the proposed references [55,56,57]. The results showed a distinguishable frequency response when the ethanol concentration was changed. Specific mixture proportions shift the frequency and amplitude signals relative to each other. The main reason for this shift was the high-loss liquid, which causes more perturbation in the radiated near field than a lower-loss fluid, and therefore, a lower resonant frequency.

## 3. Fabrication and Measurement

**Figure 11:**
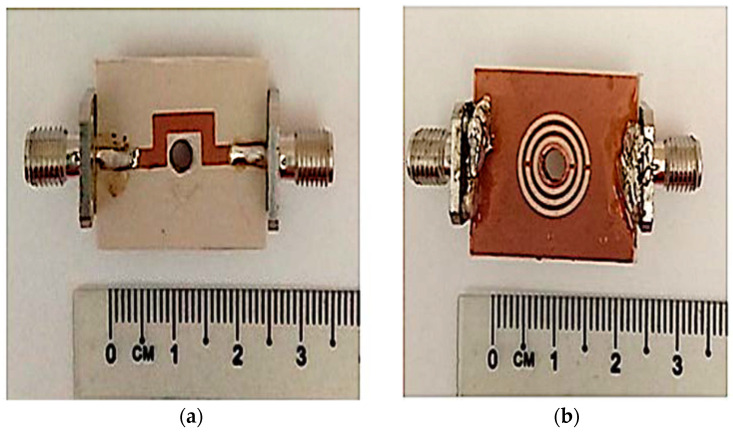
Fabricated sensor; (**a**) Front and (**b**) back perspectives.

The fabrication of the TR CSRR sensor was performed using Rogers RT Duroid 6002 substrates with a geometric width of 20 mm × 25 mm × 1.52 mm by standard photolithography and the PCB etching process. The image of the fabricated sensor is displayed in Figure 11 and its substrate has a relative permittivity 𝜀′ of 2.94 and a loss tangent 𝑡𝑎𝑛 𝛿 of 0.0012. Nevertheless, the termination between the radial 50 ohm flange connector SMA and the PCB is not good grounding, resulting in a high tolerance. Thus, it is suggested that in the future, the connector type RF with the straight 50 ohm flange attachment SMA would provide better grounding and data with minimal tolerance.

**Figure 12:**
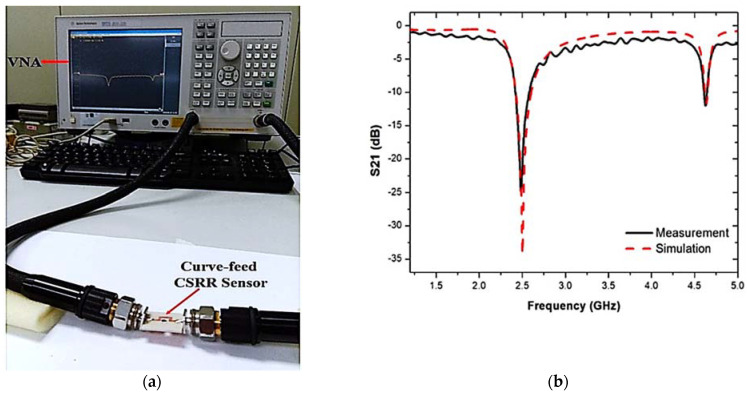
(**a**) Testing setup, and (**b**) predicated and measured outcomes of the TR CSRR sensor.

The disturbance parameters of the loaded transmission line are measured with a vector network analyzer. The response of the sensor is evaluated and recorded during the experiment when it is filled with liquid MUTs. A PP tube was incorporated into the TR CSRR sensor to evaluate the dielectric properties of the liquid samples. The experimental setup of the proposed sensor with the results of the S-parameters for the simulated and measured frequency responses is demonstrated in Figure 12.

**Figure 13:**
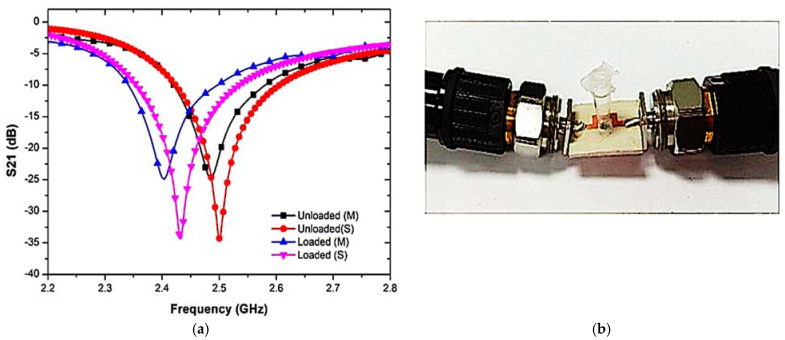
(**a**) Simulated and measured results of output load and unloaded tube as an empty sample (air), (**b**) PP tube loaded into TR CSRR sensor.

Figure 13 shows the prototype of the proposed sensor and the S-parameters of the comparison between the simulated and measured responses when the PP tube was loaded into the TR CSRR sensor. The graph shows some differences in both the simulation and measurement results, which can be attributed to fabrication errors and discrepancies between the simulated and actual parameters during the manufacturing process. These changes can affect the frequency response. The measured results clearly show the resonance frequency, quality factor, and insertion loss (S21) to be lower than those in the simulation, as tabulated in Table 3. The weak connectivity of the port couples may lead to radiation losses within the input and output port network. Therefore, simulation and manufacturing enhancements need to be investigated to minimize these errors.

Therefore, the resonance sharpness is calculated by the Q-factor. The higher the Q-factor, the narrower the resonance peak, so the sensor becomes more sensitive, with a value of 520 for the unloaded samples. In the configuration and analysis, reference materials are required for the measurements, usually used for the purpose of material characterization.

**Figure 14:**
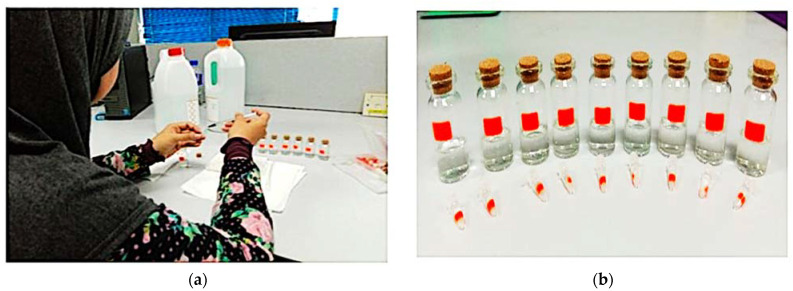
Ethanol–distilled water solutions’ preparation: (**a**) Fill the solution into a PP tube; (**b**) Ratio of the ethanol–distilled water mixture.

The preparation of the distilled water–ethanol mixing solution can be found in Figure 14. The procedural preparation of the all-around aqueous mixtures was conducted based on the total volume of the ethanol and DI water proportions. For instance, the measured quantity of an aqueous mixture was 2 mL. Of this volume, 10% was 0.2 mL of DI water and 1.8 mL was ethanol. Liquid MUTs only needed 0.3 mL for the mixture’s highest capacity to be filled into the PP tube. The samples of the liquids were manufactured by Chemiz (M) Sdn. Bhd., Malaysia. The distilled water–ethanol mixing solution (C_2_H_8_O_2_) was used to evaluate the correlation of the resonant frequency and the ethanol concentration to validate further and classify the validity of the proposed sensors. The ethanol, methanol, and distilled water samples were used in the experiment as the standard liquid sample, as they provide a wide range of permittivity for microwaves. 

Several types of liquid MUTs were used: without a tube, air (empty tube), ethanol, methanol, and DI water, which were measured to validate the sensor efficiency from 1 to 5 GHz using an Agilent Vector Network Analyzer (VNA). These liquid MUTs were loaded into a PP tube. The tube contained 7.92 μL of liquid filled by the sensing region. 

**Figure 15:**
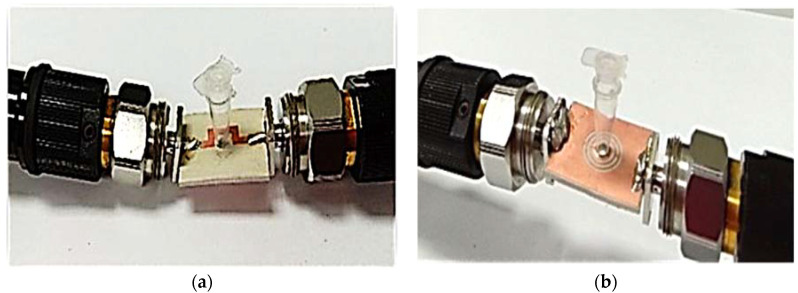
Position of PP tube loaded at the center of TR CSRR sensor; (**a**) Front and (**b**) side views.

The sample handling was also easy, and repeated analyses could be carried out efficiently. The PP tube position analysis for the liquid MUT filling was conducted before the measurement and analysis of the permittivity. It can be measured at any position, either the top or bottom of the TR CSRR sensor, as described in Figure 15.

**Figure 16:**
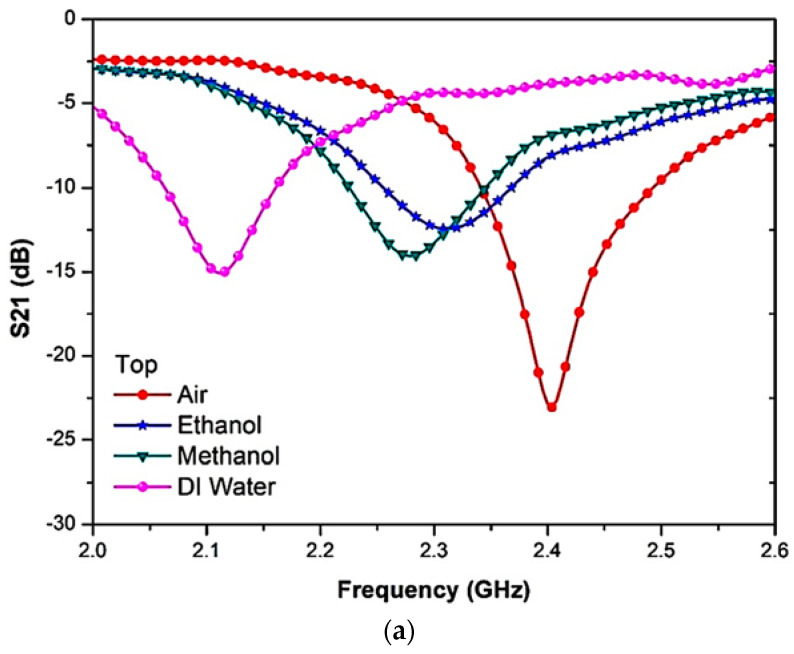

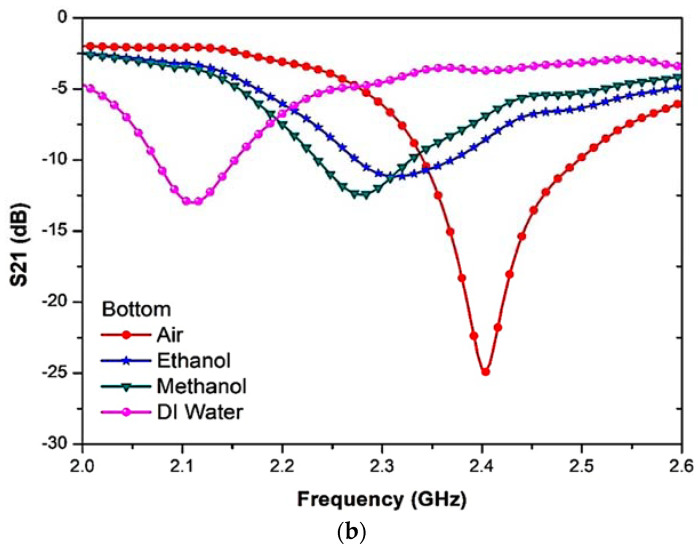
Measurement results of MUT channel location (PP tube); (**a**) Top sensor, and (**b**) Bottom Sensor.

The PP tube analysis position was conducted on the liquid and semi-solid samples, and it was noticed that the resonant frequency readings were similar. Nevertheless, there was a slight change in the amplitude at the resonant frequency. Figure 16 and Table 4 show the S-parameters for the top and bottom positions of the PP tube loaded with liquid MUTs.

## 4. Proposed Sensor Data Analysis 

### 4.1. Liquid MUTs’ Measurement and Validations

**Figure 17:**
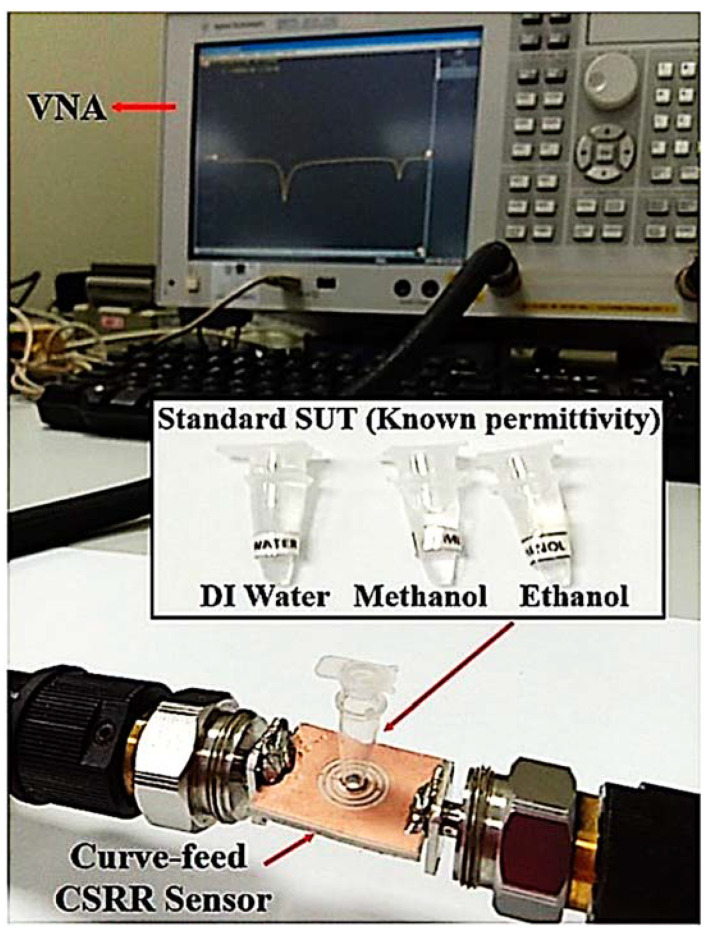
The liquid MUTs’ experimental validation for the TR CSRR sensor.

The simulation results are examined by a synthesis analysis and optimization to obtain optimal results. A comparison between the simulation and measurement results is also discussed. The study focuses on the repeatability of the details of the MUTs, complex permittivity, sensitivity, and the ethanol concentration in the MUTs samples. Other common solvents are measured (distilled water, ethanol, and methanol) to demonstrate the sensor efficiency from 1 to 5 GHz using an Agilent VNA (see Figure 17). To properly test the dielectric properties, 7.92 μL of each is required for the measurement.

**Figure 18:**
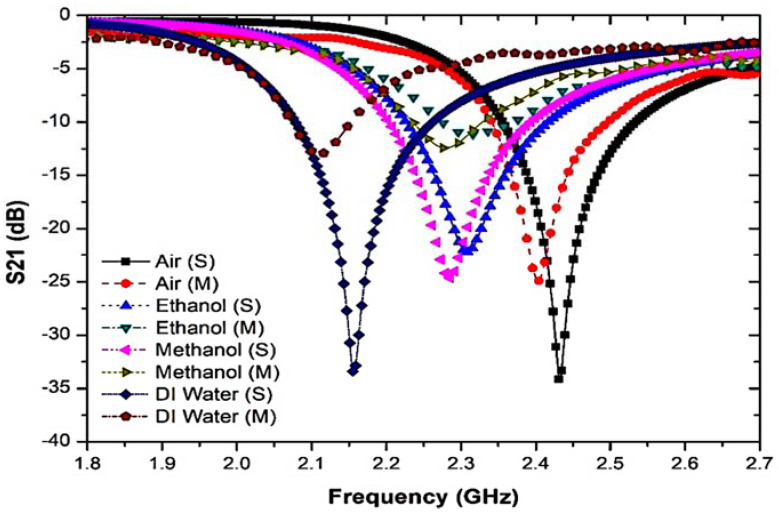
Comparison simulation and measurement of TR CSRR sensor with several solvent samples.

The values of the resonant frequency and insertion loss (S21) derived from the transmission coefficient data between the simulation and measurement outcomes are presented in Figure 18 and tabulated in Table 5.

The experimental findings indicate a strong consensus, and the simulation responses are in sequence. This might be because the empty tube is filled, and thus the resonant frequency is marginally changed to a lower frequency at 2.432 GHz by a 68 MHz shift. Nonetheless, a strong pattern of the decline in the peak amplitude of the measured outcomes leads to the lowering the sensor’s sensitivity. The shifting of the response reflects the uncertainties about the dimensions that may differ slightly during the fabrication process and the reproducibility of the port connection. 

**Figure 19:**
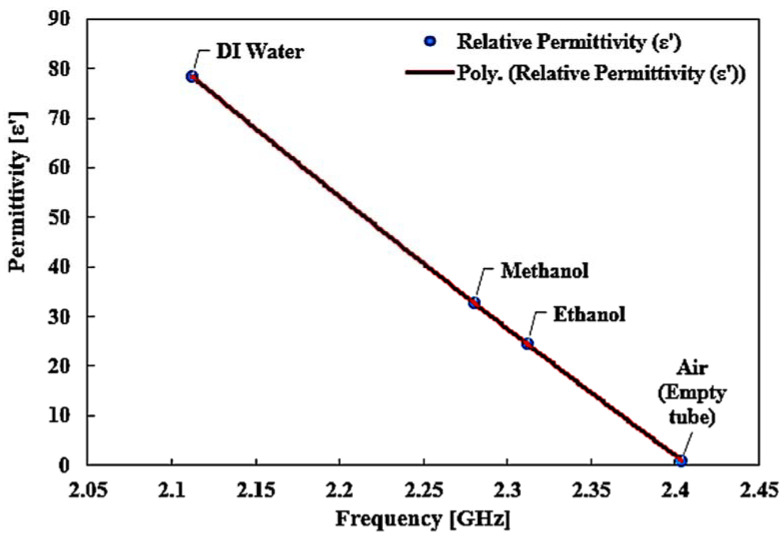
The relationship of known permittivity for materials’ properties.

Figure 19 shows the same curve-fitting technique in the second-order polynomial of MUTs of known permittivity for extracting unknown material properties. The method is used to identify the real part of the complex permittivity represented in Equation (2):

*ε*’ = 54.909𝑓^2^ − 513.31𝑓 + 917.68 (2)

**Figure 20:**
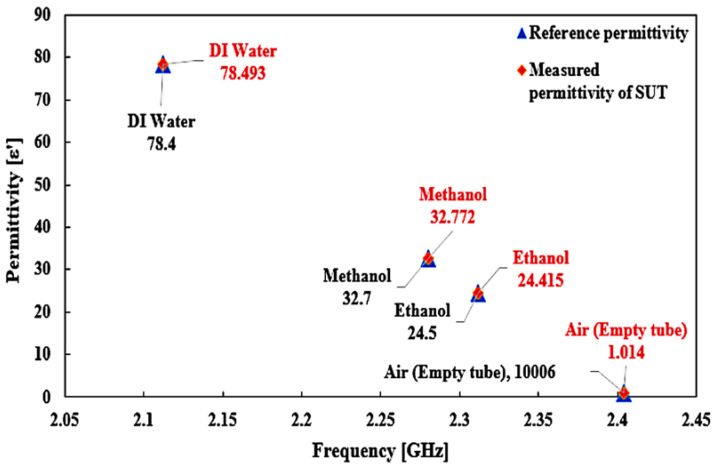
Comparison of the ideal and measured real–part permittivity of several liquid MUTs.

The comparison of the ideal and measured real–part permittivity and the percent error of various MUTs are shown in Figure 20. The experimental results and the extracted real permittivity of each MUT for the sensor are outlined in Table 6. The dielectric constant of the measured sample MUT using a TR CSRR sensor is perfectly in line with the referenced real dielectric constant for the same measurement.

The minimum and maximum error detections of the CSRR sensor are 0.119% and 1.339%, respectively, with an average tolerance of ±0.28%. This is due to the dimensional uncertainties during the production process, which cause slight deviations from the simulation model with respect to the dimensional parameters. Nevertheless, the results were lower than those of commercial dielectric probe kit sensors, which have a minimum error of 0.25% and a maximum error of 20.28%, with an average tolerance of ±0.87%. The difference in error tolerance between the two sensors is 4.99%. The main finding of the CSRR sensor is that it is more accurate and sensitive in characterizing the content of planar structures. This means that the sensor can identify and categorize aqueous solvents with a minimal volume trace.

### 4.2. Sensor Sensitivity Calculation

Considerably more analyses need to be done to indicate the performance of the proposed sensor. Sensitivity is described as the precision in differentiating between tested materials. The shifts in the resonant frequency when a MUT is introduced divided by the permittivity variation are the sensitivity of the sensor, and this can be calculated based on Equation (3) [58]:

𝑆=∆𝑓/∆*ε*′ (3)

Here, ∆𝑓 is the proportional disparity between unloaded and loaded MUTs, ∆𝑓 = (𝑓_o_ − 𝑓_s_)/𝑓_s_. At the same time, the interpretation of the dielectric constant ∆*ε* is described by the air and liquid MUTs’ dielectric constant ∆*ε*′ = (*ε*′ − (*ε*′)). To evaluate the performance of the sensor, the fractional differences in the resonant frequency for an efficient dielectric constant were measured and plotted as the sensitivity (S).

The relative differences in the rate of change of the sensor contribute to the relative change in the permittivity of the samples, which is often used as a reference for empty sample tubes (MUT = air). Table 7 displays the sensitivity of the various liquid MUT samples.

The maximum sensitivity of the TR CSRR sensor using liquid MUTs is computed to be S = 7.321 MHz/*ε_r_* or the permittivity variance. The sensor has a higher sensitivity compared to those of #1–#12 because it has larger E-fields. The presence of the electric field of the TR CSRR sensor ultimately affects the change in resonant frequency once the dielectric constant MUT is changed.

The results show that any advancement in the dielectric properties of the liquid MUTs can affect the resonant frequency, resulting in the increased sensitivity of the sensor in resonant perturbation. A comparison shows the competitive performance of the presented design in terms of compactness, Q-factor, and sensitivity, as shown in Table 8.

## 5. Conclusions

In this study, we have developed a high-efficiency microwave sensor based on the CSRR approach operating at 2.5 GHz to characterize liquid materials. We also investigated the sensor at different volumes of liquid MUTs within the PP tube and the optimal volume length was found to be 2.52 mm, which corresponds to 7.92 μL of liquid. The MUTs are filled into tubular polypropylene channels (PP) and loaded into the center hole of the CSRR resonator. The E-fields near the resonator affect the interaction with the liquid MUTs, resulting in a strong and harmonic electric field at resonance, and the measured transmission response varies significantly. Through detailed measurements, the presented TR CSRR sensor indicates some standard liquid samples and the concentrations of liquid MUT mixtures. Rogers RT Duroid 6002 was chosen as the substrate because it has low dielectric loss and stable dielectric constant with frequency. A high-frequency structure simulator (HFSS) version 15.0 was used to simulate the proposed design of the CSRR sensor. The proposed TR CSRR sensor had the best results in terms of sensitivity with a high accuracy and had a low average error detection rate of 0.23%. The sensitivity of the proposed resonator proved to be high compared to other documented sensors and is good enough to characterize three different materials with different dielectric constants easily. The final CSRR sensor has a small size, a low profile, and a high sensitivity, as well being easy to fabricate, making it a good candidate for liquid material detection.

## Figures and Tables

**Table 1:** Simulation datasets of the designed sensor with different liquid MUTs.

Liquid MUTs	Frequency (GHz)	S21 (dB)	Frequency Shift (MHz)
Without tube	2.5	−34.3829	0
Air (empty tube)	2.432	−34.3829	68
Ethanol	2.32	−22.2325	180
Methanol	2.29	−24.6268	210
DI Water	2.16	−33.6507	340

**Table 2:** Ethanol concentration analysis using TR CSRR sensor.

The Fractional Concentration of Ethanol-Water (C_2_H_8_O_2_) (%)	Resonance Frequency Shifted (GHz)	Insertion Loss Shifted (dB)
10	2.128	−14.414
20	2.144	−12.344
30	2.164	−12.737
40	2.176	−13.083
50	2.192	−13.036
60	2.212	−12.326
70	2.244	−12.698
80	2.272	−12.737
90	2.296	−12.344
100	2.312	−11.172

**Table 3:** Resonant frequency, Q–factor, and S21 magnitude (dB) simulated and measured for TR CSRR sensor.

MUT	Q-Factor	Simulation		Measurement	
		Frequency (GHz)	S21(dB)	Frequency (GHz)	S21(dB)
**Unloaded**	530	2.5	−34.281	2.484	−24.799
**Loaded**	230	2.432	−34.383	2.404	−24.901

**Table 4:** Comparative measurement results of liquid channel location (PP tube) for top and bottom TR CSRR sensor.

MUTs	Top		Bottom	
	Frequency (GHz)	S21 (dB)	Frequency (GHz)	S21 (dB)
**Air (Empty tube)**	2.404	−24.9078	2.404	−23.0683
**Ethanol**	2.312	−11.1717	2.312	−12.4376
**Methanol**	2.28	−12.4446	2.28	−14.0498
**DI water**	2.112	−13.0072	2.112	−15.0731

**Table 5:** Frequency change with various liquid MUTs samples.

MUTs	Relative Permittivity (*ε*_r_)	Simulation		Measurement	
		Frequency (GHz)	S21 (dB)	Frequency (GHz)	S21 (dB)
**Air (Empty tube)**	1.0006	2.432	−34.38	2.404	−24.90
**Ethanol**	24.5	2.32	−22.23	2.312	−11.17
**Methanol**	32.7	2.29	−24.62	2.28	−12.44
**DI water**	78.4	2.16	−33.65	2.112	−13.01

**Table 6:** Comparison of real dielectric constant and percentage error detection between the proposed and commercial sensors of several liquid MUTs.

MUT	Frequency (GHz)	Reference Real Permittivity	Real Permittivity (*ε*′)	Error (%)
Air	2.404	1.0006	1.014	1.339
Ethanol	2.312	24.5	24.415	0.347
Methanol	2.28	32.7	32.772	0.22
Water	2.112	78.4	78.493	0.119
**Average Error**			**0.28%**	

**Table 7:** Sensitivity of the several liquid MUTs.

MUTs	Frequency (GHz)	∆𝑓 (MHz)	∆𝑓	∆*ε*_r_	Sensitivity (MHz)/*ε*_r_
**Air (Empty tube)**	2.404	80	1.0006	0	0
**Ethanol**	2.312	172	24.5	23.494	7.321
**Methanol**	2.28	204	32.7	31.694	6.437
**DI Water**	2.112	372	78.4	77.394	4.807

**Table 8:** Comparison with existing works of the literature on sensors.

#	References	Sensors Sizes (mm)	Used Techniques	MUTs Samples	Frequency Band (GHz)	Q-Factor	Sensitivity (S, (MHz)/*ε_r_*))
1	[27]	80 × 40 × 0.8	Metamaterial coupling	Ethanol and Methanol	2.5	Not reported	0.27
2	[30]	80 × 25 × 0.8	Loss-compensated SRR	Glucose	1.156	190	Not reported
3	[31]	26 × 30 × 26.5	Waveguide with loop slot	Ethanol and DI water	91	Not reported	Not reported
4	[32]	112.96 × 49.16 × 3.175	Multiple split-ring resonator	Ethanol, Methanol and Air	2.1	525	Not reported
5	[59]	25 × 30 × 1.54	CCSR	Ethanol, Methanol and Milk	2.4	Not reported	Not reported
6	[60]	30 × 25 × 1.6	CSSRRs	AIR, HDPE and PVC	5.35 and 7.99	267.5	0.04
7	[61]	46 × 46 × 1.6	OCSRRs	Ethanol, Methanol and DI water	0.9	Not reported	4.3
8	[62]	28 × 20 × 0.75	CSRR	Ethanol and Water	2.85 and 2.96	145	3.0
9	[63]	35 × 25 × 1.6	SRR	Ethanol, Methanol and DI water	2.45	31	0.214
10	[64]	40× 20 × 1.6	OSRR	Ethanol, Methanol, DI water	2.5-3.5	Not reported	Not reported
11	[65]	30× 13 × 0.508	MML	Solid	5.65	217	3.25
12	[66]	38 ×35.4×15.73	GWCR	Ethanol, Methanol and Air	5.96	66.8	0.156
**This work**		**25 × 20 × 1.52**	**TR CSRR**	**Sample (without tube), Air (empty tube), Ethanol, Methanol, DI Water**	**2.5**	**520**	**7.321**

## References

[1] Lisovsky V.V. (2007). Automatic control of moisture in agricultural products by methods of microwave aqua-metry. Meas. Sci. Technol..

[2] Jónasson S.Þ., Jensen B.S., Johansen T.K. (2012). Study of split-ring resonators for use on a phar-maceutical drug capsule for microwave activated drug release. Proceedings of the 2012 42nd European Microwave Conference.

[3] Al-Gburi A.J.A., Rahman N.A., Zakaria Z., Palandoken M. (2023). Detection of Semi-Solid Materials Utilizing Triple-Rings CSRR Microwave Sensor. Sensors.

[4] Wang C., Ali L., Meng F.-Y., Adhikari K.K., Zhou Z.L., Wei Y.C., Zou D.Q., Yu H. (2021). High-Accuracy Complex Permittivity Characterization of Solid Materials Using Parallel Interdigital Capacitor- Based Planar Microwave Sensor. IEEE Sens. J..

[5] Xiang Y., Huang J., Fu L., Chen Y., Gu W., Wu Y. (2021). A Folded Substrate Integrated Waveguide Re-Entrant Cavity for Full Characterization of Magneto-Dielectric Powder Materials. IEEE Sens. J..

[6] Al-Gburi A.J.A., Zakaria Z., Rahman N.A., Alam S., Said M.A.M. (2023). A Compact and Low-Profile Curve-Feed Complementary Split-Ring Resonator Microwave Sensor for Solid Material Detection. Micromachines.

[7] Li Z., Meng Z., Soutis C., Wang P., Gibson A. (2022). Detection and analysis of metallic contaminants in dry foods using a microwave resonator sensor. Food Control.

[8] Bobowski J.S., Clements A.P. (2017). Permittivity and Conductivity Measured Using a Novel Toroidal Split-Ring Resonator. IEEE Trans. Microw. Theory Tech..

[9] Yeh C.-H., Yang C.-H. Material characterization for Zircaloy claddings in elevated temperatures using a laser ultrasound technique. Proceedings of the 2012 IEEE International Ultrasonics Symposium.

[10] Allouti N., Chausse P., Aumont C., Isselé H., Vignoud L., Rochat N., Poulain C., Gasiglia M., Sourd C., Argoud M. Photo-dielectric polymers material characterizations for 3D packaging applications. Proceedings of the 2013 IEEE 15th Electronics Packaging Technology Conference (EPTC 2013).

[11] Gao R., Yu W., Deng H., Ku H.S., Li Z., Wang M., Miao X., Lin Y., Deng C. (2022). Epitaxial titanium nitride microwave resonators: Structural, chemical, electrical, and microwave properties. Phys. Rev. Mater..

[12] Jang C., Park J.-K., Yun G.-H., Choi H.H., Lee H.-J., Yook J.-G. (2020). Radio-Frequency/Microwave Gas Sensors Using Conducting Polymer. Materials.

[13] Chernousov Y.D., Ivannikov V.I., Shebolaev I.V., Bolotov V.A., Tanashev Y.Y., Parmon V.N. (2009). Characteristics of a chemical reactor that is a loaded microwave resonator. J. Commun. Technol. Electron..

[14] Lodi M.B., Curreli N., Melis A., Garau E., Fanari F., Fedeli A., Randazzo A., Mazzarella G., Fanti A. (2021). Microwave Characterization and Modeling of the Carasau Bread Doughs During Leavening. IEEE Access.

[15] Eremenko Z.E., Ganapolskii E.M., Vasilchenko V.V. (2005). Exact-calculated resonator method for permittivity measurement of high loss liquids at millimetre wavelength. Meas. Sci. Technol..

[16] Morales-Lovera H.N., Olvera-Cervantes J.L., Perez-Ramos A.E., Corona-Chavez A., Saavedra C.E. (2022). Microstrip sensor and methodology for the determination of complex anisotropic per-mittivity using perturbation techniques. Sci. Rep..

[17] Cordoba-Erazo M.F., Weller T.M. Low-cost non-contact microwave probe design for insulating materials characterization. Proceedings of the 78th ARFTG Microwave Measurement Conference.

[18] Morozov O.G., Nasybullin A.R., Danilaev M.P., Farkhutdinov R.V. Sensor applications of Bragg microwave structures realized in coaxial waveguide. Proceedings of the 2015 International Conference on Antenna Theory and Techniques (ICATT).

[19] Yuan Z., Wang J., Zhang J., Li E., Li Y. Measurement of optical signal by Microwave Coaxial resonator. Proceedings of the 2021 IEEE MTT-S International Microwave Workshop Series on Advanced Materials and Processes for RF and THz Applications (IMWS-AMP).

[20] Mondal D., Tiwari N.K., Akhtar M.J. Microwave Assisted Non-Invasive Microfluidic Biosensor for Monitoring Glucose Concentration. Proceedings of the 2018 IEEE Sensors.

[21] Kulkarni S., Joshi M.S. (2015). Design and Analysis of Shielded Vertically Stacked Ring Resonator as Complex Permittivity Sensor for Petroleum Oils. IEEE Trans. Microw. Theory Tech..

[22] Hamzah H., Abduljabar A., Lees J., Porch A. (2018). A Compact Microwave Microfluidic Sensor Using a Re-Entrant Cavity. Sensors.

[23] Ye W., Zhao W.-S., Wang J., Wang D.-W., Wang G. A Split-Ring Resonator-Based Planar Microwave Sensor for Microfluidic Applications. Proceedings of the 2022 IEEE MTT-S International Microwave Biomedical Conference (IMBioC).

[24] Mukherjee S., Shi X., Udpa L., Udpa S., Deng Y., Chahal P. (2018). Design of a Split-Ring Resonator Sensor for Near-Field Microwave Imaging. IEEE Sens. J..

[25] Khan M.S., Varshney G., Giri P. (2021). Altering the Multimodal Resonance in Ultrathin Silicon Ring for Tunable THz Biosensing. IEEE Trans. NanoBioscience.

[26] Pan Y.M., Zheng S.Y. (2015). A Low-Profile Stacked Dielectric Resonator Antenna with High-Gain and Wide Bandwidth. IEEE Antennas Wirel. Propag. Lett..

[27] Kundal S., Khandelwal A. Highly sensitive ring resonator based refractive index sensor for label free biosensing applications. Proceedings of the 2022 International Conference on Numerical Simulation of Optoelectronic Devices (NUSOD).

[28] Bari R.T.B., Haque E., Rahman T., Faruque O. Improved Design of a Ring Resonator Based Notch Filter with High Quality Factor and Sensitivity. Proceedings of the 2022 IEEE IAS Global Conference on Emerging Technologies (GlobConET).

[29] Abdolrazzaghi M., Daneshmand M., Iyer A.K. (2018). Strongly Enhanced Sensitivity in Planar Microwave Sensors Based on Metamaterial Coupling. IEEE Trans. Microw. Theory Tech..

[30] Abdolrazzaghi M., Katchinskiy N., Elezzabi A.Y., Light P.E., Daneshmand M. (2021). Noninvasive Glucose Sensing in Aqueous Solutions Using an Active Split-Ring Resonator. IEEE Sens. J..

[31] Chudpooti N., Silavwe E., Akkaraekthalin P., Robertson I.D., Somjit N. (2017). Nano-Fluidic Millimeter-Wave Lab-on-a-Waveguide Sensor for Liquid-Mixture Characterization. IEEE Sens. J..

[32] Bahar A.A.M., Zakaria Z., Ab Rashid S.R., Isa A.A.M., Alahnomi R.A. (2017). Dielectric analysis of liquid solvents using microwave resonator sensor for high efficiency measurement. Microw. Opt. Technol. Lett..

[33] Buragohain A., Mostako A.T.T., Das G.S. (2021). Low-Cost CSRR Based Sensor for Determination of Dielectric Constant of Liquid Samples. IEEE Sens. J..

[34] Stuchly M.A., Stuchly S.S. (1980). Coaxial Line Reflection Methods for Measuring Dielectric Properties of Biological Substances at Radio and Microwave Frequencies-A Review. IEEE Trans. Instrum. Meas..

[35] Gregory A., Clarke R. (2006). A review of RF and microwave techniques for dielectric measurements on polar liquids. IEEE Trans. Dielectr. Electr. Insul..

[36] Jha S.N., Narsaiah K., Basediya A.L., Sharma R., Jaiswal P., Kumar R., Bhardwaj R. (2011). Measurement techniques and application of electrical properties for nondestructive quality evaluation of foods—A review. J. Food Sci. Technol..

[37] Gregory A.P., Clarke R.N. (2012). Tables of the Complex Permittivity of Dielectric Reference Liquids at Frequencies up to 5 GHz, NPL Report, MAT 23. https://www.researchgate.net/publication/235800733_Tables_of_the_complex_permittivity_of_dielectric_reference_liquids_at_frequencies_up_to_5_GHz.

[38] Kiani S., Rezaei P., Fakhr M. (2021). Dual-Frequency Microwave Resonant Sensor to Detect Noninvasive Glucose-Level Changes Through the Fingertip. IEEE Trans. Instrum. Meas..

[39] Navaei M., Rezaei P., Kiani S. (2022). Microwave Split Ring Resonator Sensor for Determination of the Fluids Permittivity with Measurement of Human Milk Samples. Radio Sci..

[40] Munoz-Enano J., Velez P., Gil M., Martin F. (2022). Frequency-Variation Sensors for Permittivity Measurements Based on Dumbbell-Shaped Defect Ground Structures (DB-DGS): Analytical Method and Sensitivity Analysis. IEEE Sens. J..

[41] Wu W.-J., Zhao W.-S., Wang D.-W., Yuan B., Wang G. (2022). An active microfluidic sensor based on slow-wave substrate integrated waveguide for measuring complex permittivity of liquids. Sens. Actuators A Phys..

[42] Al-Gburi A.J.A., Rahman N.A., Zakaria Z., Akbar M.F. (2023). Realizing the High Q-Factor of a CSIW Microwave Resonator Based on an MDGS for Semisolid Material Characterization. Micromachines.

[43] Kiani S., Rezaei P., Navaei M. (2020). Dual-sensing and dual-frequency microwave SRR sensor for liquid samples per-mittivity detection. Measurement.

[44] Su L., Munoz-Enano J., Velez P., Martel J., Medina F., Martin F. (2021). On the Modeling of Microstrip Lines Loaded with Dumbbell Defect-Ground-Structure (DB-DGS) and Folded DB-DGS Resonators. IEEE Access.

[45] Kandwal A., Nie Z., Igbe T., Li J., Liu Y., Liu L.W., Hao Y. (2021). Surface Plasmonic Feature Microwave Sensor with Highly Confined Fields for Aqueous-Glucose and Blood-Glucose Measurements. IEEE Trans. Instrum. Meas..

[46] Mohammadi S., Adhikari K.K., Jain M.C., Zarifi M.H. (2022). High-Resolution, Sensitivity-Enhanced Active Resonator Sensor Using Substrate-Embedded Channel for Characterizing Low-Concentration Liquid Mixtures. IEEE Trans. Microw. Theory Tech..

[47] Wiltshire B.D., Zarifi M.H. (2019). 3-D Printing Microfluidic Channels with Embedded Planar Microwave Resonators for RFID and Liquid Detection. IEEE Microw. Wirel. Components Lett..

[48] Zarifi M.H., Daneshmand M. (2016). Liquid sensing in aquatic environment using high quality planar microwave resonator. Sens. Actuators B Chem..

[49] Ansari M.A.H., Jha A.K., Akhtar M.J. (2015). Design and Application of the CSRR-Based Planar Sensor for Noninvasive Measurement of Complex Permittivity. IEEE Sens. J..

[50] Daniel R.S., Pandeeswari R., Raghavan S. (2017). Multiband monopole antenna loaded with Complementary Split Ring Resonator and C-shaped slots. AEU Int. J. Electron. Commun..

[51] Al-Gburi A.J.A., Zakaria Z., Ibrahim I.M., Aswir R.S., Alam S. (2022). Solid Characterization Utilizing Planar Microwave Resonator Sensor. Appl. Comput. Electromagn. Soc..

[52] Chuma E.L., Iano Y., Fontgalland G., Roger L.L.B. (2018). Microwave Sensor for Liquid Dielectric Characterization Based on Metamaterial Complementary Split Ring Resonator. IEEE Sens. J..

[53] Van der Eycken E.V. (2009). Practical Microwave Synthesis for Organic Chemists. J. Am. Chem. Soc..

[54] Aziz N.A.A., Malaysia U.P., Hassan J., Abbas Z., Osman N.H. (2017). Microwave Dielectric Properties of Four Types of Rhizomes from Zingiberaceace Family. J. Phys. Sci..

[55] Withayachumnankul W., Jaruwongrungsee K., Tuantranont A., Fumeaux C., Abbott D. (2013). Metamaterial-Based Microfluidic Sensor for Dielectric Characterization. Sens. Actuators A Phys..

[56] Benkhaoua L., Benhabiles M.T., Mouissat S., Riabi M.L. (2016). Miniaturized Quasi-Lumped Resonator for Dielectric Characterization of Liquid Mixtures. IEEE Sens. J..

[57] Bakir M. (2017). Electromagnetic-Based Microfluidic Sensor Applications. J. Electrochem. Soc..

[58] Jafari F.S., Ahmadi-Shokouh J. (2018). Reconfigurable microwave SIW sensor based on PBG structure for high accuracy per-mittivity characterization of industrial liquids. Sens. Actuators A Phys..

[59] Zhang X., Ruan C., Haq T.U., Chen K. (2019). High-Sensitivity Microwave Sensor for Liquid Characterization Using a Complementary Circular Spiral Resonator. Sensors.

[60] Armghan A., Alanazi T.M., Altaf A., Haq T. (2021). Characterization of Dielectric Substrates Using Dual Band Microwave Sensor. IEEE Access.

[61] Velez P., Grenier K., Mata-Contreras J., Dubuc D., Martin F. (2018). Highly-Sensitive Microwave Sensors Based on Open Complementary Split Ring Resonators (OCSRRs) for Dielectric Characterization and Solute Concentration Measurement in Liquids. IEEE Access.

[62] Wang C., Liu X., Huang Z., Yu S., Yang X., Shang X. (2022). A Sensor for Characterisation of Liquid Materials with High Permittivity and High Dielectric Loss. Sensors.

[63] Javed A., Arif A., Zubair M., Mehmood M.Q., Riaz K. (2020). A Low-Cost Multiple Complementary Split-Ring Resonator-Based Microwave Sensor for Contactless Dielectric Characterization of Liquids. IEEE Sens. J..

[64] Abdulkarim Y.I., Deng L., Karaaslan M., Altıntaş O., Awl H.N., Muhammadsharif F.F., Liao C., Unal E., Luo H. (2020). Novel Metamaterials-Based Hypersensitized Liquid Sensor Integrating Omega-Shaped Resonator with Microstrip Transmission Line. Sensors.

[65] Kiani S., Rezaei P., Navaei M., Abrishamian M.S. (2018). Microwave Sensor for Detection of Solid Material Permittivity in Single/Multilayer Samples with High Quality Factor. IEEE Sens. J..

[66] Alhegazi A., Zakaria Z., Shairi N.A., Kamarudin M.R., Alahnomi R.A., Azize A., Wan Haszerila W.H., Bahar A.A.M., Al-Gburi A.J.A. (2022). Novel Technique of Gap Waveguide Cavity Resonator Sensor with High Resolution for Liquid Detection. Int. J. Antennas Propag..

